# Divalent metal-ion transporter 1 is decreased in intestinal epithelial cells and contributes to the anemia in inflammatory bowel disease

**DOI:** 10.1038/srep16344

**Published:** 2015-11-17

**Authors:** Wei Wu, Yang Song, Chong He, Changqin Liu, Ruijin Wu, Leilei Fang, Yingzi Cong, Yinglei Miao, Zhanju Liu

**Affiliations:** 1 Department of Gastroenterology, The Shanghai Tenth People’s Hospital, Tongji University, Shanghai 200072, China; 2 Departments of Microbiology and Immunology, The University of Texas Medical Branch, Galveston, TX 77555, USA; 3 Department of Gastroenterology, The First Affiliated Hospital of Kunming Medical University, Kunming 650032, China

## Abstract

Divalent metal-ion transporter 1 (DMT1) has been found to play an important role in the iron metabolism and hemogenesis. However, little is known about the potential role of DMT1 in the pathogenesis of anemia from patients with inflammatory bowel disease (IBD). Herein, we investigated expression of DMT1 in the intestinal mucosa by quantitative real-time polymerase chain reaction (qRT-PCR) and immunohistochemistry, and found that DMT1 was significantly decreased in the inflamed mucosa of active IBD patients compared with that in those patients at remission stage and healthy controls. To further study the mechanism, we cultured HCT 116 cell line *in vitro*. Expression of DMT1 in HCT116 was demonstrated to be markedly decreased under stimulation with TNF for 24 and 48 h, while JNK inhibitor (JNK-IN-7) could significantly reverse the decrease. Interestingly, anti-TNF therapy successfully improved anemia in clinical responsive Crohn’s disease patients, and DMT1 was found to be markedly up-regulated in intestinal mucosa. Taken together, our studies demonstrate that decreased expression of DMT1 in intestinal mucosa leads to compromised absorption and transportation of iron and that blockade of TNF could rescue anemia and promote DMT1 expression in gut mucosa. This work provides a therapeutic approach in the management of anemia in IBD.

Crohn’s disease (CD) and ulcerative colitis (UC) are two major disease subjects of inflammatory bowel disease (IBD). Although the exact etiology is still not completely understood, IBD appears to be disorders of the host immune response to luminal microbiota which involves in a state of local immune hyper-reactivity^1^,^2^,^3^,^4^. Patients with IBD often suffer from abdominal pain, diarrhea and chronic fatigue. These symptoms significantly impact on the quality of patients’ life. Chronic fatigue is frequently overlooked and caused by anemia^5^. Bergamaschi *et al.* have reported that 104 of 263 patients with IBD were anemic and that this kind of anemia was not influenced by age, gender and azathioprine treatment^6^. The prevalence and severity of anemia are relevant to the activity of the bowel disorder. Anemia in IBD patients may result from several causes such as lack of iron and other chronic diseases. Some patients are also caused by loss of vitamin B12 and folate, drug-associated side-effects (e.g., sulfasalazine, thiopurine) and several hematologic diseases^7^,^8^,^9^. Consistent with these findings, intravenous iron therapy has been performed in the treatment of anemia in patients with IBD and proven to be effective in the clinic^10^.

Among the etiologies of anemia in IBD including iron deficiency anemia (IDA) and anemia of chronic disease (ACD), one common feature is an abnormal iron status^11^. In humans, the majority of iron absorption occurs in the upper digestive tract through intestinal epithelial cells or enterocytes^12^. Apart from ferritin and transferrin, some proteins such as divalent metal-ion transporter 1 (DMT1), ferroportin 1 (FPN1), duodenal cytochrome b (DcytB) and hephaestin (HP) may also take part in these processes^13^.

DMT1, also named as SLC11A2, divalent cation transporter1 (DCT1), and Nramp2, is the main iron uptake transporter which mediates the transport of ferrous iron across the brush border membrane of intestinal epithelial cells^14^, and functions as the main iron uptake transporter^15^. Another transporter protein heme carrier protein 1 (HCP1) which enables transmembrane transport of haem molecules into enterocytes is also involved in iron absorption^16^. Previous study has demonstrated that intestinal inflammation interferes iron metabolism and expression of DMT1^17^. Abnormality of iron metabolism influences the level of erythropoietic activity. Interestingly, Srivastava *et al.* have found that DMT1 may play a role in the pathogenesis of CD^18^. However, it is still unknown whether DMT1 is involved in the anemia of IBD.

Tumor necrosis factor (TNF) is produced as a soluble or transmembrane protein by lamina propria mononuclear cells. After binding to its receptors TNFR1 and TNFR2, TNF exerts various pro-inflammatory functions in colitis followed by the activation of the transcription factor nuclear factor-κB (NF-κB)^19^. There are many proteins involved in the TNF signaling such as c-Jun N-terminal kinase (JNK), caspase-3 and caspase-8. Previous work has shown that TNF could down-regulate DMT1 expression in Caco-2 cells after 72 h of TNF exposure^20^ and contribute to ACD by reduced duodenal iron transfer in mice^21^.

In this study, we investigated the levels of hemoglobin (Hb), serum iron, ferritin, transferrin, folic acid, vitamin B12, and DMT1 in patients with IBD, and explored the potential role of DMT1 in the anemia of disease. We found that anemia was frequently present in patients with IBD. The level of serum iron was quite lower in anemic patients and DMT1 was significantly decreased in the inflamed mucosa of the patients with active IBD compared with that in these patients at remission stage and healthy controls. The TNF-JNK pathway was also found to play a vital role in DMT1 expression *in vitro*. TNF could down-regulate DMT1 expression, while JNK inhibitor (JNK-IN-7) could markedly suppress this function. Importantly, anti-TNF mAb therapy (infliximab, IFX) could markedly improve anemia in patients with active CD^22^,^23^. Thus, our study provides evidence that DMT1 is the critical protein involved in the pathogenesis of anemia of IBD patients and may serve as a potential therapeutic target in the treatment of anemia in IBD.

## Results

### Anemia is frequently present in IBD patients

**Figure 1:**
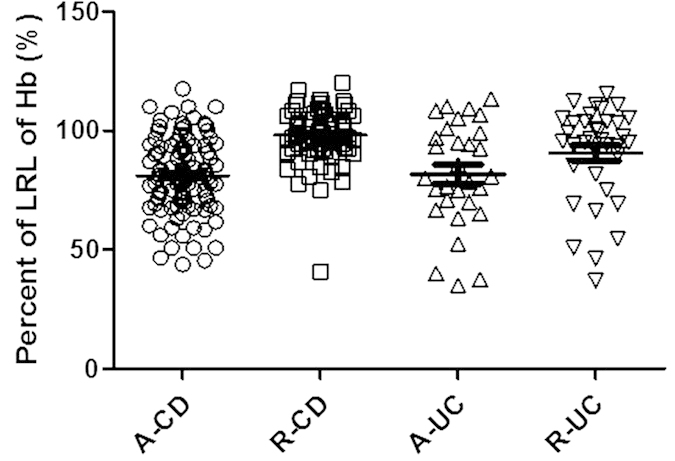
Anemia is frequently present in IBD patients. The level of Hb less than 120.0 g/L in non-pregnant women or 130.0 g/L in men is defined as the LRL of Hb. The level of Hb at the LRL was arbitrarily set to 100%, while relative changes of Hb concentrations in IBD were expressed as percent as compared with the LRL. A-CD (active CD, n = 102), R-CD (CD in remission, n = 77), A-UC (active UC, n = 30), R-UC (UC in remission, n = 36). Data were expressed as mean ± SEM.

Previous work has reported that anemia frequently occurs in IBD patients. To further study anemia in Chinese IBD patients, the level of Hb less than 120.0 g/L in non-pregnant women and less than 130.0 g/L in men is defined as anemia^24^. Here we used the ratio of patients’ Hb to the lower range limit (LRL) to describe the degree of anemia. We identified 245 IBD patients (including 179 patients with CD and 66 with UC) and 30 healthy controls. Among the 245 patients, 171 people suffered from anemia including 126 CD and 45 UC. If the level of Hb at the LRL was arbitrarily set to 100%, relative changes of Hb concentrations in IBD were expressed as percent as compared with the LRL. The percent in the patients with active CD (A-CD) was 81.04 ± 1.59%, and 98.16 ± 1.29% in CD patients in remission (R-CD), respectively. While the percents were 81.77 ± 4.01% in active UC (A-UC) versus 90.67 ± 3.30% in UC with remission (R-UC), respectively (Fig. 1).

**Figure 2:**
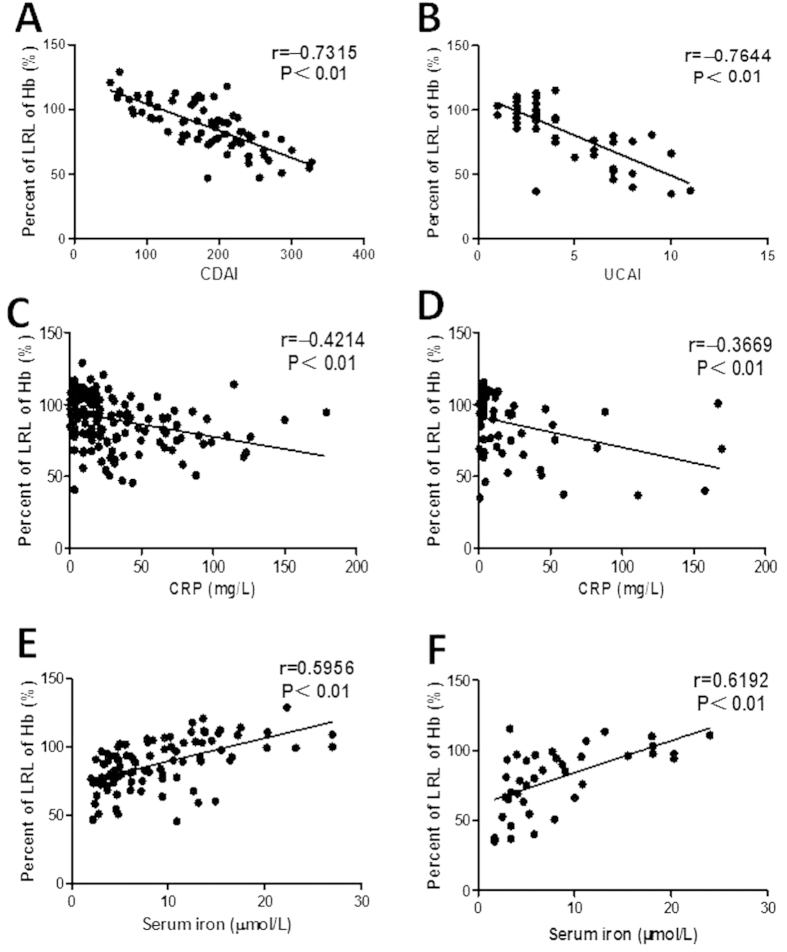
The severities of anemia are relevant to the disease activity, CRP, and serum iron, respectively. Relative changes of serum Hb concentrations were expressed as percent as compared with the LRL. A and B, Correlations between CDAI or UCAI and percents of LRL of Hb in CD ((**A**) n = 71) or UC ((**B**) n = 50) patients, respectively. (**C**) Correlation between CRP and percents of LRL of Hb in CD patients (n = 179). (**D**) Correlation between CRP and percents of LRL of Hb in UC patients (n = 66). (**E**,**F**) Correlations between the levels of serum iron and percents of LRL of Hb in CD ((**E**) n = 98) and UC ((**F**) n = 39) patients, respectively. Spearman’s correlation analysis was performed. *P* values are shown in each panel.

Crohn’s disease activity index (CDAI), Sutherland disease activity index for UC (UCAI) and C-reactive protein (CRP) of the IBD patients are normally used to evaluate the disease activity. As illustrated in Fig. 2A, there was a negative correlation between CDAI and percentage of the LRL of Hb in CD patients (*r* = −0.7315, *P* < 0.01). A negative correlation between UCAI and percentage of the LRL of Hb was also detected in UC patients (Fig. 2B; *r* = −0.7644, *P* < 0.01). Moreover, a weaker negative correlation was still found between the levels of CRP and percentage of the LRL of Hb in CD and UC patients, respectively (*r* = −0.4214, *P* < 0.01 in CD; *r* = −0.3669, *P* < 0.01 in UC) (Fig. 2C,D). These data illustrate that anemia does frequently occur in IBD patients and is closely associated with the disease activity.

### Changes of biochemical parameters in IBD patients

As shown in Table 1, the mean serum iron concentrations were 9.37 ± 0.82, 8.19 ± 0.96, and 16.54 ± 3.78 μmol/L in A-CD, A-UC patients and healthy controls, respectively. Serum iron levels in active IBD patients were significantly decreased than healthy controls (*P* = 0.004 in CD, *P* = 0.0038 in UC). A strong positive correlation was observed between serum iron levels and percent of the LRL of Hb in A-CD or A-UC patients (*r* = 0.5956, *P* < 0.01 in A-CD; *r* = 0.6192, *P* < 0.01 in A-UC) (Fig. 2E,F). However, there was no such a significant difference in serum levels of vitamin B12, folic acid, ferritin and transferrin between IBD patients and healthy controls (*P* > 0.05). These results indicate that the majority of IBD patients with anemia suffer from serum iron deficiency, consistent with previous reports^7^,^8^,^9^.

### MRNA levels of DMT1, FPN1, DctyB and HP in inflamed mucosa of IBD

**Figure 3:**
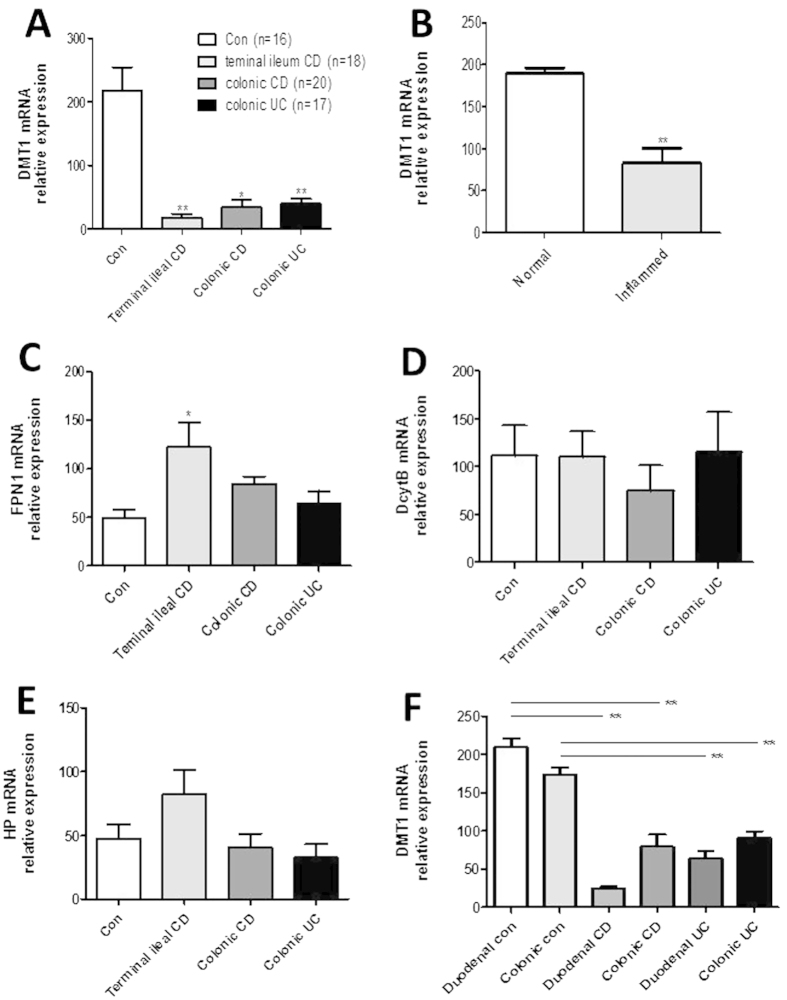
mRNA expression of DMT1, FPN1, DcytB and HP in intestinal mucosa. The levels of mRNA of DMT1 (**A**), FPN1(**C**), DcytB (**D**) and HP (**E**) were determined in intestinal mucosa by qRT-PCR (controls: n = 16, terminal ileum CD: n = 18, colonic CD: n = 20, colonic UC: n = 17). (**B**) Paired intestinal mucosal biopsies were taken from normal or inflamed mucosa from the same patients with A-CD (n = 12), and mRNA levels of DMT1 were analyzed by qRT-PCR. (**F**) Biopsies were taken from 15 patients with CD, 11 patients with UC, 10 healthy controls. The levels of mRNA for DMT1 were detected by qRT-PCR. Gene expression was normalized to GAPDH mRNA levels in each sample. (**A**,**D**,**E**) Kruskal-Wallis test, (**B**) Student’s *t* test, (**C**,**F**) one way ANOVA. ^*^P < 0.05, ^**^P < 0.01 versus controls (Con). Data are expressed as mean ± SEM. The data are a representative of 3 independent experiments.

Since DMT1, FPN1, DctyB, and HP have been found to involve in the iron metabolism, we then analyzed the mRNA expression in the intestinal mucosa from patients with IBD and healthy controls by qRT-PCR. As shown in Fig. 3A, relative expression of DMT1 was significantly decreased in inflamed ileum from patients with CD (*P* < 0.01), inflamed colon from patients with CD (*P* < 0.05) and inflamed colon from patients with UC (*P* < 0.01) compared with that from healthy controls. Moreover, DMT1 expression was also found to be decreased in inflamed mucosa compared with normal areas from the same patients of CD (Fig. 3B) (*P* < 0.01). In contrast, the levels of FPN1 expression were significantly increased in terminal ileum of CD patients compared with healthy controls (*P* < 0.05) (Fig. 3C). However, there was no difference in expression of DctyB and HP in intestinal mucosa between IBD patients and healthy controls (Fig. 3D,E). Moreover, expression of FPN1, DcytB and HP was also measured in both inflamed and normal mucosa from the same patients with CD, no difference was detected (supplementary Fig. 1). Thus, these results identify that DMT1 is markedly reduced in the inflamed mucosa of patients with IBD. It may eventually play a role in the etiopathology of anemia in IBD patients by influencing the iron absorption.

Previous data have suggested that DMT1 is mainly expressed on duodenal enterocytes, while another work reported that DMT1 is also expressed in the cortex of kidney present at the brush border and apical pole of epithelial cells of proximal tubules^25^. To further analyze the mRNA expression of DMT1 at different parts of intestine, we collected intestinal biopsies from duodenum and colon of patients with A-CD or A-UC and healthy controls. The results revealed that levels of DMT1 mRNA were also significantly decreased in duodenum from patients with A-CD or A-UC compared with normal controls (*P* < 0.01) (Fig. 3F). Moreover, expression of DMT1 mRNA was also found to be decreased in inflamed colon of patients with A-CD or A-UC compared with healthy controls (*P* < 0.01) (Fig. 3F), while no difference was observed between duodenum and colon from the same patients (*P* > 0.05). Therefore, it is supposed that DMT1 is expressed in both duodenal and colonic mucosa and that colonic enterocytes may also act slightly on the absorption of dietary iron.

### *In situ* expression of DMT1 in inflamed mucosa of IBD

**Figure 4:**
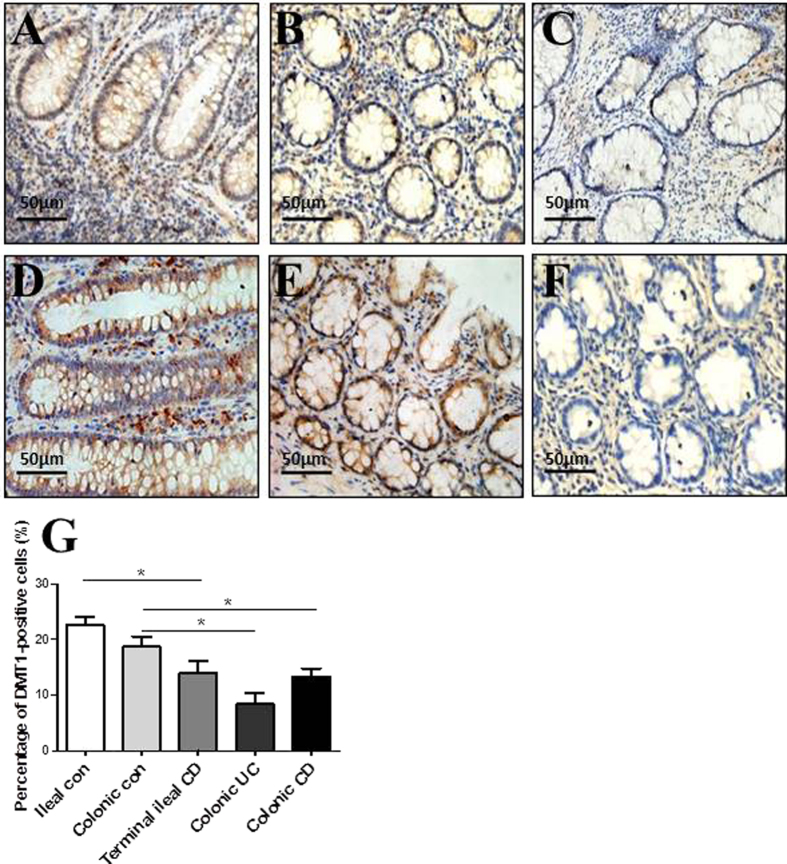
*In situ* expression of DMT1 in intestinal mucosa. Representative frozen sections were prepared from inflamed mucosa of a patient with terminal ileal CD (**A**), a patient with colonic CD (**B**), a patient with colonic UC (**C**), a healthy ileal control (**D**), a healthy colonic control (**E**), and negative control (**F**) by immunohistochemistry (original magnification, ×400). (**G**) Quantification of DMT1-positive cells in the intestinal mucosa of ileal control, colonic control, terminal ileal CD, colonic UC and colonic CD. The percentage of positive cells was calculated as follows: [(positive cells)/(total cells)] × 100. Data are expressed as mean values of positive cells per high power field ± SEM of 3 independent experiments. One way ANOVA was performed. ^*^P < 0.05.

Immunohistochemical staining was performed to determine *in situ* expression of DMT1 in intestinal mucosa from patients with IBD and normal controls. As shown in Fig. 4, DMT1-positive cells were observed to be significantly decreased in the inflamed mucosa of ileal CD (Fig. 4A), colonic CD (Fig. 4B) and colonic UC (Fig. 4C) compared with normal ileal and colonic mucosa (Fig. 4D,E). The majority of DMT1-positive cells were intestinal epithelial cells and some lamina propria mononuclear cells. These positive cells manifested a dot-like pattern or membrane-staining pattern, while a small amount of positive cells exhibited punctuate paranuclear staining. The percentage of DMT1-positive cells in terminal ileal CD (14.00 ± 2.08%) was significantly lower than that in ileal controls (22.64 ± 1.45%) (*P* < 0.05). Moreover, the percentage in colonic UC (8.33 ± 2.03%) and colonic CD (13.34 ± 1.45%) was also significantly lower than that in colonic controls (18.67 ± 1.86%) (*P* < 0.01 in UC, *P* < 0.05 in CD). No marked difference of DMT1 positivity was observed between ileal CD and ileal UC or colonic CD and colonic UC (*P* > 0.05) (Fig. 4G). These results further confirm that DMT-1 is markedly decreased in inflamed mucosa of IBD patients.

### TNF down-regulates DMT-1 expression in intestinal epithelial cells *in vitro*, but is rescued by JNK inhibitor

**Figure 5:**
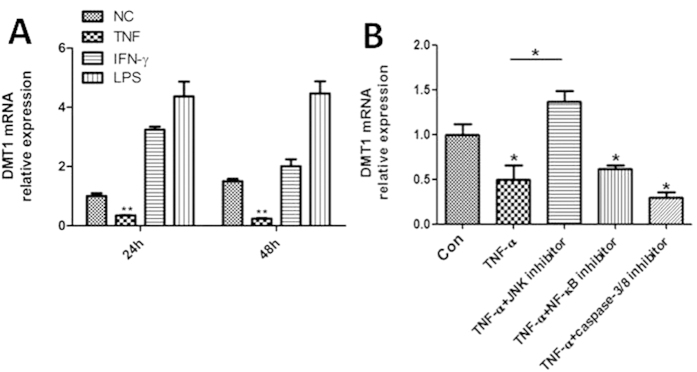
TNF down-regulates the DMT-1 expression *in vitro*, but rescued by JNK inhibitor. (**A**) HCT116 cell line was cultured and stimulated with TNF (20 ng/mL), LPS (100 ng/mL) and IFN-γ (20 ng/mL) *in vitro*. Cells were collected after 24 and 48 h of culture, and mRNA levels of DMT1 expression were then determined by qRT-PCR. One way ANOVA was performed for statistical analysis. ^**^P < 0.01 versus the data of medium alone (NC). (**B**) HCT116 cells were incubated with TNF (20 ng/mL) in the presence of JNK inhibitor (JNK-IN-7, 1 μM), NF-κB inhibitor (BAY 11-7082, 1 μM), and caspase-3/8 inhibitor (Z-DEVD-FMK, 50 μM), respectively, as indicated. Cells were then harvested after 48 h of culture, and DMT1 mRNA expression was then determined by qRT-PCR. One way ANOVA was performed for statistical analysis. ^*^P < 0.05 versus the data of control group. Data are expressed as mean ± SEM. Gene expression was normalized to GAPDH mRNA levels in each sample. The data are a representative of 3 independent experiments.

We then investigated the mechanisms involved in decreased DMT1 expression in inflamed intestinal mucosa of IBD patients. Accumulating evidence has shown that TNF and IFN-γ are markedly increased in inflamed intestinal mucosa of IBD patients compared to those in healthy controls, and that lipopolysaccharide (LPS) could also induce inflammatory response *in vitro*. To determine whether these inflammatory mediators were responsible for the down-regulation of DMT1, the epithelial cell line (HCT116 cell) was cultured *in vitro* and stimulated with LPS, TNF and IFN-γ, respectively. The results demonstrated that TNF significantly down-regulated DMT1 mRNA expression after 24 and 48 h of culture (*P* < 0.01), while LPS and IFN-γ did not have such an effect (Fig. 5A). To further determine the mechanisms underlying TNF involved in DMT1 expression, various inhibitors in the TNF signaling pathways were selected and co-incubated with HCT116 *in vitro*. As shown in Fig. 5B, co-incubation of JNK signaling inhibitor (JNK-IN-7) significantly suppressed the down-regulation of the DMT1 mRNA expression (*P* < 0.05), while TNF alone or co-incubation with NF-κB and caspase-3/8 inhibitors significantly down-regulated DMT1 mRNA expression compared to controls (*P* < 0.05). No significant difference was observed between control group and JNK-IN-7 treated group. Thus, our results prove that TNF could down-regulate DMT1 expression through the JNK pathway.

### Anti-TNF treatment increased DMT1 mRNA expression in clinical responsive CD patients

**Figure 6:**
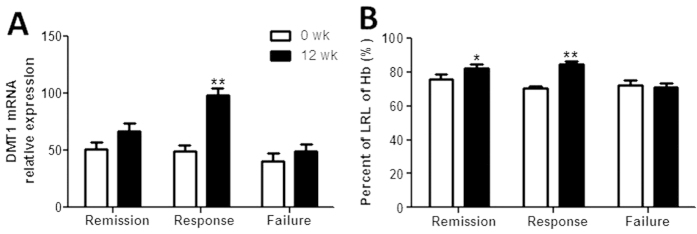
Anti-TNF mAb therapy promotes DMT1 expression in intestinal mucosa and improves anemia in CD patients. Active CD patients (n = 72) received treatment with anti-TNF mAb (IFX) as indicated, and the disease activity and efficacy of IFX therapy were assessed 12 weeks after the first infusion. (**A**) Intestinal biopsies were taken from these CD patients 12 weeks after the first IFX infusion, and the mRNA levels of DMT1 were detected by qRT-PCR. Gene expression was normalized to GAPDH mRNA levels in each sample. (**B**) Sera were collected from these CD patients before and weeks 12 after the first IFX infusion. Hb was then analyzed and the percent of the LRL of Hb was compared. Data are expressed as mean ± SEM. Mann-Whitney test was used for statistical analysis. ^*^P < 0.05, ^**^P < 0.01 versus the data before therapy. The data are a representative of 3 independent experiments. Remission (clinical remission group, n = 38), response (clinical response group, n = 20), failure (clinical failure group, n = 14).

To further investigate the potential role of TNF in the pathogenesis of anemia of IBD, 72 patients with active CD were treated with anti-TNF mAb (IFX) as described previously^22^,^23^. Anemic analysis was performed in these CD patients before and week 12 after the first infusion of IFX. As shown in Fig. 6A, expression of DMT1 mRNA was demonstrated to be markedly increased after IFX treatment in clinical response group (*P* < 0.01). Importantly, Fig. 6B shows that the percentage of the LRL of Hb was significantly increased 12 weeks after IFX treatment in both the clinical remission (*P* < 0.05) and response groups (*P* < 0.01). Taken together, these data prove that TNF plays a role in the pathogenesis of anemia in IBD through blocking the DMT1 expression.

## Discussion

Anemia is a common complication in most patients with IBD. Previous work has demonstrated that a prevalence of anemia in IBD patients is 21% to 27% in European countries and that 57% of anemic patients are iron deficient^26^. Different pathogenic mechanisms have been reported to be involved in the cause of anemia in IBD, including anemia of chronic disease, iron deficiency, chronic intestinal blood loss, vitamin B12 and folic acid^27^. In this study, we first determined the level of Hb was decreased in Chinese IBD patients, and found that there was a significantly negative relationship between CRP and Hb, CDAI or UCAI and Hb in patients with CD and UC. To investigate the mechanisms of anemia in IBD patients, we found that serum iron was also decreased and had a strong positive correlation with level of Hb. Furthermore, we also observed that anemia was due to the decrease of DMT1 in inflamed mucosa, the latter was down-regulated by TNF but rescued by JNK inhibitor (JNK-IN-7). Therefore, our results reveal that intestinal inflammation-induced anemia is associated with DMT1 expression which regulates the metabolism of iron.

Several causes associated with hemogenesis and anemia have been determined in our study. There was a strong positive correlation between Hb and serum iron in both CD and UC patients, but not between Hb and folate or vitamin B12, although previous work has shown that anemia in CD may result in lack of vitamin B12 and folate^28^,^29^. These results suggest that majority of the anemic IBD patients suffer from abnormal iron metabolism. Recent studies have demonstrated that IDA and ACD are two most common causes of anemia in IBD^30^. Increasing lines of evidence have demonstrated that ACD is very common in IBD patients and that many factors such as pro-inflammatory cytokines or other immune disorders may contribute to the occurrence of ACD^31^. Actually, the level of serum ferritin should be decreased in IDA compared with healthy controls, but it does not happen in ACD. In our data, we have observed that there was no significant difference in levels of ferritin and transferrin between IBD patients and healthy controls. All these evidences support a notion that the popularity of ACD is present in IBD patients.

Under physiological conditions, three vital cells participate in iron homeostasis, including duodenal enterocytes absorbing iron, macrophage recycling iron, and hepatocytes storing iron. In general, there are two ways for absorbing dietary iron. One is transferrin-transferrin receptor pathway. Iron reaching to the basolateral surface of the enterocyte is rapidly bound to transferrin, an iron-binding protein. Transferrin then binds to transferrin receptor to deliver iron. Another way is several proteins involved, including DcytB which is a ferric reductase, DMT1 which is an important ferrous iron importer, FPN1, and ferroxidase HP for transporting iron. In general, ferric iron can be reduced to ferrous form by DcytB on the apical or brush border membrane. When iron enters into the enterocyte, it can be stored as ferritin or transported into blood circulation. Interestingly, DMT1 is found to be significantly reduced in the inflamed mucosa of the patients with active IBD compared with that at remission stage and healthy controls, suggesting that low serum iron may be caused by the decrease of DMT1^14^,^32^,^33^.

TNF as an important pro-inflammatory cytokine plays a vital role in the pathogenesis of IBD. TNF signaling also makes pleiotropic pro-inflammatory effects such as augmented angiogenesis, the induction of Paneth cell death, the production of matrix metalloproteinases, the activation of macrophages and effector T cells and the direct damage of intestinal epithelial cells^19^. In our study, we found DMT1 expression was significantly decreased after TNF exposure, consistent with previous report. Interestingly, JNK-IN-7 significantly suppressed the down-regulation of the DMT1 expression. Recent work has demonstrated that membrane-bound TNF, rather than soluble TNF, acts on intestinal inflammation and that neutralization of membrane-bound TNF with specific antibody such as IFX could induce T cell apoptosis and suppress experimental colitis in mice^34^. In our study, we showed that clinical responsive CD patients after anti-TNF treatment had high levels of serum Hb and DMT1 mRNA in intestinal mucosa than those before therapy. In our recent study^22^, Hb as one kind of parameter reflecting the nutritional status was found to be apparently improved after IFX treatment in CD patients from clinical response and remission groups. Therefore, a hypothesis could be envisaged in that IFX therapy could restore intestinal epithelial barrier, leading to enhanced absorption of iron. Additionally, it is also supposed that TNF could induce the anemia in IBD patients by blocking the DMT1 expression.

In conclusion, our study demonstrates that DMT1 is involved in the development of anemia in IBD and that regulation of DMT1 may become a potential therapeutic approach in the anemia treatment of IBD.

## Methods

### Ethical considerations

This study was approved by the Institutional Review Board for Clinical Research of the Shanghai Tenth People’s Hospital of Tongji University and the methods were carried out in accordance with the approved guidelines. Informed consent was also obtained from all subjects.

### Patients and sample collection

All patients with IBD were recruited from the Department of Gastroenterology, the Shanghai Tenth People’s Hospital of Tongji University (Shanghai, China) from January 2012 to May 2015. The diagnosis of IBD was based on the conventional clinical, radiological and endoscopic features, and finally confirmed by histological examination of ileal and colonic biopsies. Blood samples were taken from 179 patients with CD, and 66 patients with UC (108 males and 137 females, age 15–81 yrs). All patients had not received hormone, iron, biological agents, and blood transfusion therapy in recent one month and did not suffer from any other autoimmune diseases (such as ankylosing spondylitis, rheumatoid arthritis, autoimmune hepatitis), infectious diseases (such as acute infectious enteritis, intestinal tuberculosis, intestinal amebic dysentery) and malignant diseases. Clinical characteristics of patients with IBD and controls are shown in Table 2. Additionally, blood samples were also obtained from 30 healthy donors (15 men and 15 women, age 25–48 yrs) for comparison.

Inflamed ileal and/or colonic tissues were collected from 38 patients with CD (18 in ileum, 20 in colon) and 17 patients with UC who underwent endoscopy (24 men and 31 women, age 18–65 yrs). Biopsies were taken at sites of active inflammation adjacent to ulcerations or normal mucosa according to the experiment requirement. The macroscopically and microscopically unaffected ileal and colonic mucosa were also collected from 16 patients with colon carcinoma (8 men and 8 women, age 38 –57 yrs) who underwent colectomy and served as controls.

### Analysis of biochemical parameters

Blood samples were obtained from both IBD patients and healthy controls after overnight fasting using a vacuum tube. Determination of Hb, serum iron, ferritin, transferrin, folic acid, vitamin B12 and C-reactive protein (CRP), was performed according to routine laboratory tests (Beckman, Brea, CA, USA).

### Reagents

The RNeasy Kit was purchased from Qiagen (Valencia, CA, USA). SYBR PrimeScript RT reagent kits were purchased from TaKaRa (Dalian, China). Envision flex peroxidase-blocking reagent was purchased from Dako (Glostrup, Denmark). Mouse anti-human DMT1 mAb was purchased from Abcam (Cambridge, MA, USA). Horseradish peroxidase (HRP)-conjugated rabbit anti-mouse IgG was purchased from Jackson Immunoresearch Laboratories (West Grove, PA, USA). Dulbecco’s Modified Eagle Medium (DMEM), fetal bovine serum (FBS), penicillin (100 U/mL) and streptomycin (100 g/mL), L-gentamycin, and 2-ME were all purchased from HyClone (Logan, UT, USA). The inhibitor of JNK and caspase-3/8 were purchased from Selleckchem (Shanghai, China). The inhibitor of NF-κB was purchased from Sigma-Aldrich (St. Louis, MO, USA).

### Quantitative real-time polymerase chain reaction (qRT-PCR)

Fresh-frozen biopsies were obtained from 16 healthy controls, 18 terminal ileal CD patients, 20 colonic CD patients, and 17 colonic UC patients to determine mRNA expression of DMT1, FPN1, DcytB, and HP. Paired inflamed and normal mucosal biopsies were obtained from 12 CD patients to verify DMT1 mRNA expression. Duodenal and colonic biopsies from 10 healthy controls, 15 patients with CD and 11 patients with UC were also collected for DMT1 mRNA expression. Total RNA was extracted from the fresh-frozen biopsies using the RNeasy Kit according to the manufacturer’s instructions. The quantity and quality was assessed using a NanoVue spectrophotometer (GE Healthcare, Piscataway, NJ, USA), with a 260/280 ratio of >1.8 and 28S/18S ratio of >1.4 for the majority of the samples. The complementary DNA was synthesized with the TaKaRa SYBR PrimeScript reverse RT reagent kit, according to the manufacturer’s instructions. Reverse transcription-PCR reactions were performed using the following conditions: 25 °C for 10 min, 42 °C for 15 min, followed by 85 °C for 5 min. The synthesized cDNA was stored in refrigerator at −20 °C. Quantitative real-time PCR was performed in the ABI prism 7900 HT sequence detector (Applied Biosystems, Foster City, CA, USA) using SYBR green methodology. GAPDH was used as the endogenous reference gene. All primers were synthesized from ShengGong BioTeck (Shanghai, China) and used according to standard methodologies. PCR reactions were performed with 40 cycles using the following conditions: 95 °C for 1 min, followed by 40 cycles at 95 °C for 15 s and 60 °C for 30 s. All samples for qRT-PCR analysis were performed in triplicate wells. The relative levels of target gene expression were calculated as a ratio relative to the GAPDH reference mRNA. Quantitative real-time PCR analysis was carried out as described previously^23^,^35^,^36^. The specific primers were synthesized as follows: DMT1 (sense, 5′-CTAGACTGGGAGTGGTTACTGG-3′, antisense, 5′-AGGATGACTCGTGGGACCTT-3′); FPN1 (sense, 5′- TGGATGGGTTCTCACTTCCTG-3′, antisense, 5′- GTCAATCCTTCGTATTGTGGCAT-3′); Dcytb (sense, 5′-CAGCACTTATGGGATTGACAGAG-3′, antisense, 5′-CCTTCTGGCGGGAATGTACTG-3′); HP (sense, 5′-TGCAGGCACTCTACAAGGTC-3′, antisense, 5′-AGGCTTCATATCGCACTTTCC-3′); GAPDH (sense, 5′-TCCTCATGCCTT CTTGCCTCTTGT-3′, antisense, 5′-AGGCGCCCAATACGACCAAATCTA-3′). (ShengGong Biotech, Shanghai, China).

### Immunohistochemistry

Immunohistochemistry was performed on 5-μm-thick sections from fresh-frozen intestinal mucosal tissues from IBD patients and control individuals. Sections were air-dried overnight, fixed in acetone for 10 min and rinsed in phosphate-buffered saline (PBS) for 5 min. After incubation with Envision flex peroxidase-blocking reagent for 10 min, these sections were then incubated with mouse anti-DMT1 mAb (dilution 1:200) at 4 °C overnight. After washing, the sections were incubated for 60 min with HRP-conjugated rabbit anti-mouse IgG (dilution 1:400) at room temperature. The color reaction was developed with 3,3′-diaminobenzidine and the sections were counterstained with haematoxylin. Pre-experiments were performed before the formal experiments to normalize the whole staining procedures. As negative controls which could ensure that the IHC staining is sensitive and specific, sections were treated with PBS instead of primary antibody^37^,^38^. The positive cells and total cells were subsequently counted in high power fields from all samples. The percentage of positive cells was calculated as follows: [(positive cells)/(total cells)] × 100.

### Culture of intestinal epithelial cell line

Intestinal epithelial cell line (HCT116) was cultured in DMEM medium, supplemented with 10% heat-inactivated fetal bovine serum (FBS), penicillin (100 U/mL) and streptomycin (100 g/mL), 2 mM L-gentamycin, and 50 μM 2-ME. These cells were stimulated with TNF (20 ng/mL), LPS (100 ng/mL), and IFN-γ (20 ng/mL), respectively. After 24 or 48 h of culture, cells were harvested followed by extraction of total RNA, and the levels of DMT1 mRNA were analyzed by qRT-PCR. To determine the mechanisms of TNF involved in regulating DMT1 expression, JNK inhibitor (JNK-IN-7, 1 μM), NF-κB inhibitor (BAY 11-7082, 1 μM), and caspase-3/8 inhibitor (Z-DEVD-FMK, 50 μM) were also added into the culture medium. After 48 h of culture, cells were then collected to detect the expression of DMT1 by qRT-PCR.

### Anti-TNF treatment

Seventy-two patients with active CD (43 males and 29 females, age 19–46 yrs with mean 29.5 ± 5.9 yrs) were treated with anti-TNF mAb (5 mg/kg, IFX; Cilag AG, Schaffhausen, Switzerland) at weeks 0, 2, and 6 as described previously^22^,^23^. IFX was administrated according to the manufacture’s instruction, and infusions were delivered over a two-hour period. These patients were monitored weekly during the follow-up, serum samples were collected at week 12 after the first infusion. Clinical remission was defined as a CDAI score of <150 points, and clinical response as a decrease in the CDAI score ≥70 points at the evaluation time point compared to the baseline index. The failure category included all the remaining patients, whose CDAI was not significantly changed or increased. Colonoscopy was performed prior to and 12 weeks after initial therapy of IFX, and mucosal lesion status was then evaluated according to the Simple Endoscopic Score for Crohn’s disease (SES-CD) score 0 to 3 of every five ileocolonic segments. Endoscopic remission was assessed as a SES-CD score of 0 to 2. Intestinal biopsies were collected at sites of active inflammation adjacent to ulcerations for histology and analysis of the mRNA levels of DMT1 by quantitative real-time PCR as indicated above.

### Statistical analyses

All data were expressed as mean ± standard error of the mean (S.E.M.). Statistical analysis was performed using SPSS statistics version 14.0 (SPSS, Chicago, IL, USA). Statistical significance was calculated by unpaired Student’s *t* test, Mann-Whitney test, Kruskal-Wallis test and one-way analysis of variance (ANOVA). Correlation was analyzed by Spearman’s correlation analysis. A value of *P* < 0.05 was considered statistically significant.

## Additional Information

**How to cite this article**: Wu, W. *et al.* Divalent metal-ion transporter 1 is decreased in intestinal epithelial cells and contributes to the anemia in inflammatory bowel disease. *Sci. Rep.* **5**, 16344; doi: 10.1038/srep16344 (2015).

## Supplementary Material

Supplementary Information

## Figures and Tables

**Table 1:** Prevalence of serum iron, vitamin B12 and folic acid in IBD patients.

Group	Serum iron (μmol/L)	Folic acid (ng/ml)	Vitamin B12 (pmol/L)	Ferritin (ng/ml)	Transferrin (g/L)
CD (n = 179)	9.37 ± 0.82^+^	8.65 ± 0.71	380.20 ± 24.80	191.40 ± 30.67	2.86 ± 0.87
Anemia (n = 126)	7.45 ± 0.90^*^	8.27 ± 0.82	389.4 ± 29.36	202.8 ± 38.21	3.01 ± 1.12
No anemia (n = 53)	15.68 ± 1.19	10.14 ± 1.37	344.2 ± 40.90	147.60 ± 20.87	2.35 ± 0.09
UC (n = 66)	8.19 ± 0.96 ±	8.80 ± 0.68	478.10 ± 49.03	164.70 ± 30.09	2.09 ± 0.15
Anemia (n = 45)	6.67 ± 0.84^*^	8.35 ± 0.68	488.10 ± 59.92	131.40 ± 29.25	1.98 ± 0.17
No anemia (n = 21)	15.11 ± 2.51	10.75 ± 2.09	436.80 ± 48.67	312.50 ± 83.10	2.60 ± 0.26
Healthy Con. (n = 30)	16.54 ± 3.78	10.90 ± 1.87	325.83 ± 37.90	123.85 ± 28.81	2.78 ± 0.21

^+^P < 0.005 vs healthy controls; ^*^P < 0.001 vs no anemic patients in the same group.

**Table 2:** Clinical characteristics of patients with IBD and controls.

	Biopsy samples			Blood samples		
	Con	CD	UC	Con	CD	UC
Numbers of patients	16	38	17	30	179	66
Age (y)	46.1 ± 6.9	41.3 ± 16.8	34.6 ± 13.3	37.2 ± 9.8	42.6 ± 11.2	38.7 ± 10.3
Gender						
Male	8	17	7	15	64	44
Female	8	21	10	15	115	22
Disease duration (month)		43.2 ± 17.2	49.2 ± 23.5		32.5 ± 22.1	39.6 ± 20.2
Current therapy						
5-aminosalicylates		32	11		153	51
Immunosuppressants		0	0		0	0
Biologics		0	0		0	0
Nutritional therapy		2	0		7	4
Disease extent (UC)*						
E1			4			8
E2			7			28
E3			6			30
Disease location (CD)*						
L1		9			37	
L2		13			45	
L3		16			97	
L4		0			0	

^*^According to the Montreal classification system.
