# Supplementary material for: Divalent metal-ion transporter 1 is decreased in intestinal epithelial cells and contributes to the anemia in inflammatory bowel disease
Source: Sci Rep. 2015 Nov 17;5:16344. doi: 10.1038/srep16344 (PMC4648093; doi:10.1038/srep16344)
Supplement: Supplementary Information [file srep16344-s1.doc]

**Divalent metal-ion transporter 1 is decreased in intestinal epithelial cells and contributes to the anemia in inflammatory bowel disease**

Wei Wu^1*^, Yang Song^1*^, Chong He^1*^, Changqin Liu^1^, Ruijin Wu^1^, Leilei Fang^1^, Yingzi Cong^2^, Yinglei Miao^3^ & Zhanju Liu^1^

^1^Department of Gastroenterology, The Shanghai Tenth People’s Hospital, Tongji University, Shanghai 200072, China. ^2^Departments of Microbiology and Immunology, The University of Texas Medical Branch, Galveston, TX 77555, USA. ^3^Department of Gastroenterology, The First Affiliated Hospital of Kunming Medical University, Kunming 650032, China.

**Supplementary Figure 1| Expression of FPN1, DcytB and HP mRNA in inflamed mucosa compared with normal areas from the same CD patients.** Paired intestinal mucosal biopsies were taken from normal or inflamed mucosa from the same patients with active CD (n = 12), and mRNA levels of FPN1 (A), DcytB (B) and HP (C) were analyzed by qRT-PCR. Gene expression was normalized to GAPDH mRNA levels in each sample. Data are expressed as mean ± SEM. The data are a representative of 3 independent experiments.


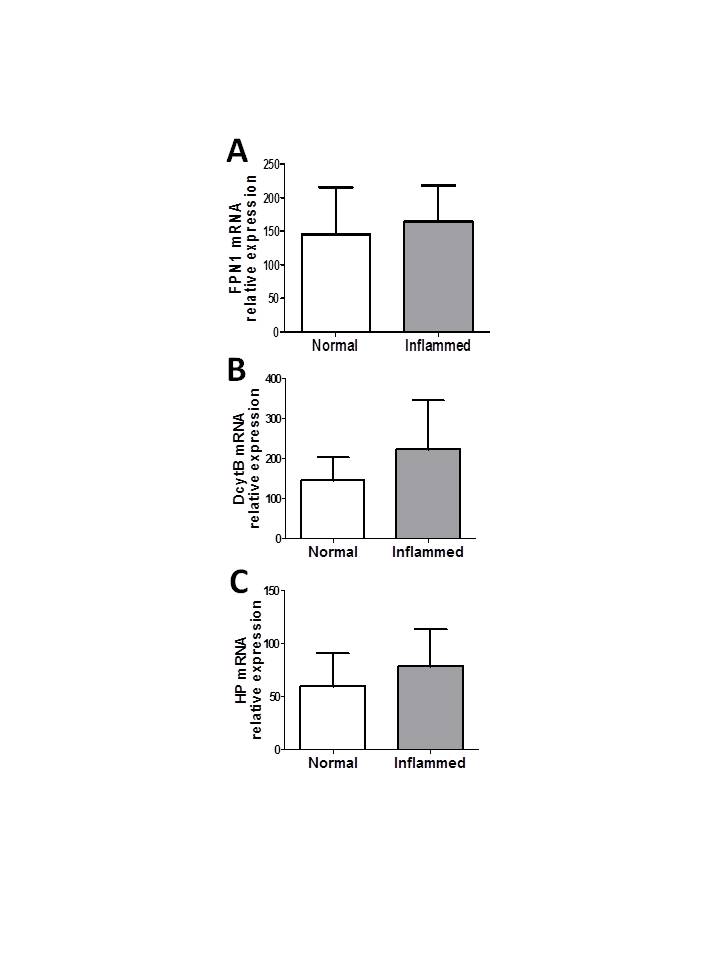
**Supplementary Figure 1**
